# Bridging the Gap Between Training and Reality: A Dental Scientist Trainee Perspective

**DOI:** 10.1002/jdd.70038

**Published:** 2025-09-10

**Authors:** Kazune Pax, Seung Jin Jang, Caris M. Smith, Nicholas G. Fischer, Kristina Astleford‐Hopper, Jeremie O. Piña, Rachel J. Kulchar, Drashty P. Mody, Shawn A. Hallett

**Affiliations:** ^1^ Division of Oral Biosciences College of Dentistry The Ohio State University Columbus Ohio USA; ^2^ Department of Oral Biology University of Florida College of Dentistry Gainesville Florida USA; ^3^ Department of Oral and Maxillofacial Surgery Institute of Oral Health Research School of Dentistry University of Alabama at Birmingham Birmingham Alabama USA; ^4^ MDRCBB‐Minnesota Dental Research Center for Biomaterials and Biomechanics University of Minnesota Minneapolis Minnesota USA; ^5^ Department of Diagnostic and Biological Sciences University of Minnesota Minneapolis Minnesota USA; ^6^ Section on Craniofacial Genetic Disorders Eunice Kennedy Shriver National Institute of Child Health and Human Development (NICHD), National Institutes of Health (NIH) Bethesda Maryland USA; ^7^ School of Dentistry University of Maryland Baltimore Maryland USA; ^8^ University of California Los Angeles School of Dentistry Los Angeles California USA; ^9^ Department of Cariology Indiana University School of Dentistry Indianapolis Indiana USA; ^10^ Department of Periodontics and Oral Medicine University of Michigan School of Dentistry Ann Arbor Michigan USA; ^11^ Department of Hematology/Oncology Boston Children's Hospital Boston Massachusetts USA

**Keywords:** advancement, dentist‐scientist, education, research, reform

## Abstract

Dental schools stand at a crossroads. While research drives clinical innovation and improves patient outcomes, the pipeline for training future dentist‐scientists remains underdeveloped. Programs such as DDS/DMD‐PhD pathways and NIDCR‐supported initiatives aim to integrate scientific inquiry with clinical training. However, students pursuing academic careers continue to encounter a complex set of systemic challenges, including burnout, financial hardship, and an academic culture that undervalues basic research contributions. Structural disparities between DDS/DMD‐PhD and MD‐PhD pathways highlight the lack of robust postdoctoral opportunities and sustained mentorship within dental academia. DDS/DMD‐PhD trainees often graduate into a professional landscape that offers limited viable options for balancing independent research with clinical responsibilities. Without intentional reforms, dentistry risks losing a generation of motivated, research‐oriented clinicians who can bridge the widening gap between science and practice. In this perspective, we outline key barriers encountered by DDS/DMD‐PhD students across the United States and call for cultural and structural changes. These include enhanced financial support, clearer career trajectories, and greater institutional commitment to academic dentistry. Ensuring the future of dental research and education requires not just training dentist‐scientists, but creating an environment where they are equipped, supported, and inspired to lead.

## 1 Introduction

Research is not just important to dentistry, it is the foundation upon which the entire profession stands. Without a continuous stream of scientific discovery, dentistry cannot advance, adapt to emerging health challenges, or fulfill its mandate to patient care. This concept relies on sustained and rigorous research. Dental, oral, and craniofacial research have uncovered clear connections between oral and systemic health, such as the notion that periodontal diseases are linked to diabetes, cardiovascular disease, adverse pregnancy outcomes, and glaucoma [1]. To stay at the forefront of care, dentistry must invest in clinician‐scientists who will generate new knowledge, translate discoveries into clinical practice, and train the next generation of dentists and oral health researchers [2]. Failing to do so risks relegating dentistry to a static, isolated discipline rather than the dynamic, integrated field it has become. Research is not a supplement to dental education and practice, it is the engine that powers it forward.

## 2 Paving the Path Forward for Dentist‐Scientist Trainees

**FIGURE 1:**
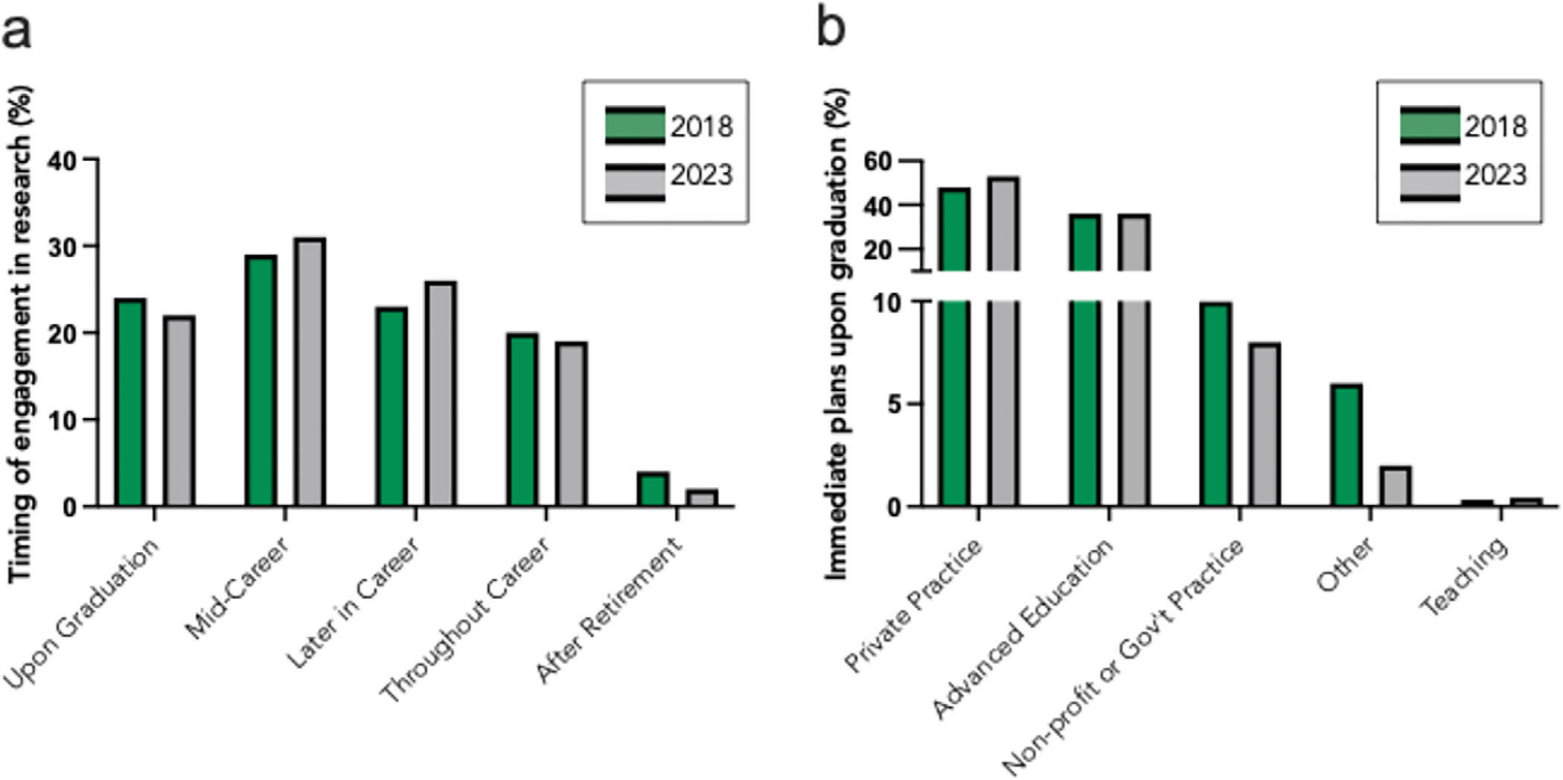
The United States senior dental engagement in research and immediate career plans after graduation in 2018 and 2023. Data are obtained from Istrate et al. [4] who collected data from senior predoctoral students from accredited US dental schools using a repeated, cross‐sectional survey. Approximately 48% of senior students in 2023 responded, compared with 65% in 2018 across 65 of the 66 accredited US dental schools. (a) Timing of engagement in research activity during their professional career for the 20% of graduating students that indicated interest in engaging in research activities. (b) Immediate professional plans upon graduation. “Other” refers to positions related to dentistry that were not included in the survey, non‐dentistry related positions, and unsure professional plans. “Teaching” refers to working at a dental school as a clinical and/or didactic educator immediately upon graduation.

Our collective experiences and opinions as DDS/DMD‐PhD students and recent graduates across the country highlight a deep and widespread barrier to pursuing academic careers: burnout. This challenge is not anecdotal; it is systemic. Burnout among trainees is characterized by persistent mental exhaustion, a diminished sense of personal accomplishment, significant financial strain, and pervasive uncertainty about long‐term career viability [3]. When investigating the likelihood of pursuing a career in research, the American Dental Education Association (ADEA) Survey of Dental School Seniors determined the most common career stage for becoming involved in research was mid‐career or later, increasing from 52% of seniors in 2018 to 57% in 2023 (Figure 1a) [4]. This coincides with limited interest in academic teaching immediately following graduation as just 0.33% and 0.43% of respondents in 2018 and 2023, respectively, showed interest (Figure 1b). Although the reasons for research involvement at these stages were not explicitly defined, we believe this timeline allows one to hone their clinical repertoire immediately following completion of dental school and then transition into research investigation once those skills are established.

The 2017 ADEA Survey of Dental School Seniors showed that, upon graduation, only 0.4% of graduating students planned to enter the academic workforce, despite 58% indicating an interest in teaching. Indeed, 48.3% of graduates will enter dental private practice and 36.9% will pursue specialty clinical training [5], further emphasizing a gap between one's interest and subsequent commitment to pursuing academic dentistry. Analysis of vacant faculty positions at US dental schools from 2005 to 2016 revealed that, in more recent years, retirement has emerged as a greater challenge to faculty retention than competition with the private sector [6]. In the same study, over 40% of dental faculty members surveyed were over 60 years old. Similar trends exist across other dental research‐intensive countries (personal communication with faculties from Australia, Canada, China, Israel, Sweden, and the United Kingdom). The landscape of training pathways is diverse; therefore, we must determine methods to inspire students to remain in research to make significant impacts within the entire oral health research community.

Acknowledging these challenges, the National Institute of Dental and Craniofacial Research (NIDCR), alongside research‐oriented dental institutions, have initiated dentist‐scientist training programs with the goal of bridging the gap between clinical practice and research. DDS/DMD‐PhD training supplements a traditional 4‐year dental curriculum with 3–4 years dedicated to doctoral research. Dual degree programs are designed to integrate deep understanding of dental practice with basic, translational, and clinical research. Programmatic structures vary across institutions, with several placing dental school first followed by doctoral research [7], while others sandwich doctoral research between 4 years of clinical training, a structure commonly found in MD/PhD programs. However, this format makes reentry into clinical practice challenging following a 4‐year hiatus committed to one's research.

Many programs are supported by institutional training grants (i.e. T32/T90) managed by the NIDCR, which provides most financial support for dual degree students. In addition, the K12 award supports dentists to combine specialty clinical training with doctoral research. The Medical Scientist Partnership Program (MSPP) and Medical Research Scholars Program (MRSP) at the NIDCR Intramural Campus provide additional opportunities for dental students to supplement their clinical training with short‐term research experience under the supervision of NIDCR intramural investigators. The culmination of these NIDCR‐supported initiatives emphasizes mentored research, professional development, and a multidisciplinary approach to addressing complex oral and systemic health questions.

It is our collective opinion, although not qualitatively measured, to recognize the inevitability of burnout and feelings of lostness among students. The pressures of achieving clinical proficiency and scientific impact, with an impending graduation date, require a supportive educational environment that fosters resilience, encourages work‐life balance, and promotes student well‐being. Importantly, structuring postdoctoral pathways to offer robust academic and research career opportunities that foster independent thought is vital. In the following, we highlight the need for strategic interventions and support systems to enhance educational and professional development of future dentist‐scientists.

MD‐PhD programs have demonstrated a remarkable ability to retain graduates within academic (67%), industry (8%), and research‐oriented (4%) roles following graduation, showcasing their effectiveness in preparing students for diverse career paths in biomedical fields [8]. In contrast, approximating data from Herzog et al., DDS/DMD‐PhD programs have struggled to replicate these outcomes as 47.5% of graduates pursue an academic position, 8.5% in industry, and 44.1% in private practice [9]. Moreover, MD‐PhD graduates benefit from a multitude of postgraduate routes that better blend clinical practice and biomedical research, paving the way for successful physician‐scientist careers [8]. These avenues encompass residency programs tightly intertwined with research in medical fields, alongside subspecialty fellowship programs, fostering, fulfilling, and intellectually enriching career trajectories. In contrast, DDS/DMD‐PhD graduates encounter a dearth of established postgraduate pathways that effectively merge dental clinical practice with biomedical research in dental, oral, and craniofacial fields. The options available to DDS/DMD‐PhD graduates often involve costly dental specialty residencies or postdoctoral research positions that may not fully utilize their clinical training. This disparity underscores the urgent need for structural and cultural reforms within dental academia to create more cohesive and gratifying postgraduate avenues, aligning with the ambitions of these driven individuals.

Financial constraints further compound the challenges faced by DDS/DMD‐PhD students. Even those fortunate enough to receive full‐ride scholarships, which includes tuition support and an annual stipend, students may encounter unexpected expenses such as instrument leasing fees, significantly reducing their stipends. Based on our personal experiences, dentist‐scientist predoctoral trainees live on an income according to the NIH predoctoral guidelines of $28,788 (2025) per year for the duration of their 7‐to 8‐year programs [10]. They grapple with a stark financial reality, especially when considering that the annual cost of living for a single individual in the United States, excluding rent, is approximately $14,060 (data from Numbeo). Further, most US dental institutions are based in cities, where costs of living are significantly higher than rural areas. Despite their impressive research accolades—evidenced by NIH/NIDCR F30 fellowships, Student Competition for Advancing Dental Research and its Application (SCADA), and IADR/AADOCR Hatton awards—the financial support for attending meetings such as AADOCR/IADR is inadequate. Based on our collective experiences across many research‐intensive dental schools, support is often limited to an allocated budget from T32 or F30 grants with minimal or no additional support from institutions. However, we recognize that these opinions do not represent the financial limitations of all dentist‐scientist training programs. Indeed, the student dues for AADOCR and IADR often use majority of the support provided by the T32 or F30 grants, underscoring the financial challenges students face to share their research and make connections.

Considering these challenges, another question arises: how does a student remain involved in academia and research postgraduation? Many students begin this career path because they were interested in the intersection between research and clinical care. In addition, our time to graduation has often presented a major hurdle as there are few clear paths that allow a blend of both research and clinical care. Moreover, we have also observed through personal interactions with senior peers, that dentist‐scientist graduates who choose not to pursue specialty training face limited options as hiring a specialist into a specific dental school department traditionally offers fewer logistical issues. Conducting research as a general dentist in an academic setting is difficult due to demanding teaching responsibilities and relatively low salaries, making it difficult to sustain a career in research without additional institutional support or external sources of funding. Moreover, based on interactions with senior peers and our own personal experiences, due to the overwhelming time commitment associated with postdoctoral research and clinical involvement, simultaneous completion of these two paths is rare and burdensome. For students pursuing specialty training, programs that integrate residency and research, such as the K12 Dental Specialty and PhD Program pathway supported by the NIDCR, offer a promising path forward. However, this opportunity poses significant challenges, including navigating complex administrative processes and obtaining the grant itself. Despite the existence of such pathways, it is through our learned experiences in predoctoral teaching clinics, that program directors and clinical faculty are often marked by skepticism in students attempting to blend research and clinical work. These attitudes highlight the systemic barriers that continue to hinder the seamless integration of clinical and research training for dual degree students.

Despite evidence suggesting that individuals pursuing a PhD after completing their dental education are more likely to remain in academia [7], restructuring the current DDS/DMD‐PhD dual degree programs to programs that combine post‐DDS dental specialty residency and PhD training may not be the most effective solution. As combined residency training and PhD programs expand, resident‐PhD trainees face additional challenges, such as annual student loan payments from undergraduate and dental educations that commonly exceed the maximum amount covered by the NIH Loan Repayment Program. If dental specialty residency programs modeled medical residency and fellowship programs, where a salary covering living expenses is offered, there may be greater financially feasible options available to keep graduates in academia. However, due to financial constraints at institutional and government levels, most resident‐PhD trainees incur additional costs not covered by grant mechanisms alone. Lastly, a great hurdle experienced by those pursuing combined clinical specialty and PhD training is dedicated time to independent research. The unwavering pressure from clinical programs for their students to excel at clinical skills leaves little time for intensive research investigation. Thus, those who effectively combine both must do so with complete support from both clinical and scientific mentorship and leadership.

## 3 Conclusion

Collectively, this perspective prompts a critical examination of the structural and cultural changes needed within dental academia to better support and realize the aspirations of ambitious DDS/DMD‐PhD students. Examining the differences between MD‐PhD and DDS/DMD‐PhD programs and postgraduate opportunities may provide insight to begin reforming the DDS/DMD‐PhD trajectory. From our adapted analysis of Istrate et al. [4], exploring ways in which graduating dental students can become more committed to the pursuit of careers in research immediately following their graduation is critical to ensuring a strong future academic dental workforce (Figure 1). For example, exposing first and second year dental students to basic, clinical, and translational research through formal mentored research programs may inspire interest which can be built upon during the latter phases of their dental education. In addition, supporting the training of dentist‐scientists from governmental, public, and private institutions, but also mentors and advocates, is imperative to ensuring the longitudinal success of academic dental institutions and the advancement of dental, oral, and craniofacial research. Students yearn to enter the academic workforce, as demonstrated by our commitment to several years of independent scientific research that in many cases overlaps with their clinical training. However, daunting challenges make it difficult to persevere. A growing environment of skepticism in science are compounded by current challenges faced by the NIH. To truly bridge the gap between training and reality, the dental profession must invest in systems that value and sustain the dual commitment to science and patient care. If we hope to secure the future of dental innovation, we must champion DDS/DMD‐PhD trainees not as outliers, but as essential architects of academic dentistry's next chapter.

## Author Contributions

K.P., S.J.J., C.M.S., N.G.F., K.A.H., J.O.P, R.J.K., D.P.M., and S.A.H. contributed to data acquisition, analysis, and interpretation, and drafted the manuscript. K.P., N.G.F., and S.A.H. contributed to conception and critically revised the manuscript. All authors gave final approval and agreed to be accountable for all aspects of the work.

## Conflicts of Interest

The authors declare no conflicts of interest.
